# Selective Modulators of PPAR-*γ* Activity: Molecular Aspects Related to Obesity and Side-Effects

**DOI:** 10.1155/2007/32696

**Published:** 2007-01-08

**Authors:** Fang Zhang, Brian E. Lavan, Francine M. Gregoire

**Affiliations:** Department of Biology, Metabolex Inc., 3876 Bay Center Place, Hayward, CA 94545, USA

## Abstract

Peroxisome proliferator-activated receptor *γ* (PPAR-*γ*) is a key regulator of lipid metabolism and energy balance implicated in the development of insulin resistance and obesity. The identification of putative natural and synthetic ligands and activators of PPAR-*γ* has helped to unravel the molecular basis of its function, including molecular details regarding ligand binding, conformational changes of the receptor, and cofactor binding, leading to the emergence of the concept of selective PPAR-*γ* modulators (SPPAR*γ*Ms). SPPAR*γ*Ms bind in distinct manners to the ligand-binding pocket of PPAR-*γ*, leading to alternative receptor conformations, differential cofactor recruitment/displacement, differential gene expression, and ultimately differential biological responses. Based on this concept, new and improved antidiabetic agents for the treatment of diabetes are in development. This review summarizes the current knowledge on the mechanism of action and biological effects of recently characterized SPPAR*γ*Ms, including metaglidasen/halofenate, PA-082, and the angiotensin receptor antagonists, recently characterized as a new class of SPPAR*γ*Ms.

## 1. INTRODUCTION

PPAR-*γ* belongs to the nuclear receptor superfamily and is a member of the NR1C subgroup that includes PPAR-*α* and PPAR-*δ*. These receptors form heterodimers with the retinoid X receptor (RXR), bind to PPAR response elements (PPREs) in the regulatory region of target genes, and modulate their transcription. PPAR-*γ* is expressed most abundantly in adipose tissue and is a master regulator of adipogenesis. PPAR-*γ* activation promotes adipocyte differentiation and is associated with induction of lipogenic enzymes and glucoregulatory molecules. PPAR-*γ* ligands include a surprisingly diverse set of natural ligands [1], such as prostaglandin PGJ2, linolenic, eicosapentaenoic, docohexaenoic, and arachidonic acids, and synthetic ligands, such as the thiazolidinediones (TZDs), L-tyrosine-based compounds, several nonsteroidal anti-inflammatory drugs (NSAIDs), and a variety of new chemical classes.

The clinical relevance of PPAR-*γ* is highlighted by the currently marketed antidiabetic blockbuster drugs, rosiglitazone (Avandia), and pioglitazone (Actos). These antidiabetic drugs of the TZD class behave as potent and selective PPAR-*γ* full agonists [2]. In humans, they enhance insulin action, improve glycemic control with a significant reduction in the level of glycated haemoglobin (HbA_1C_), and have variable effects on serum triglyceride levels in patients with type 2 diabetes [3, 4]. Despite their proven efficacy and widespread use, these drugs possess a number of deleterious side effects, including significant weight gain and peripheral edema [5].

The weight gain associated with the use of TZDs is due to multiple interacting factors. Because these agents promote adipocyte differentiation and lipid storage [6], increased adiposity is likely to be a major cause of the observed weight gain. Several studies have indeed shown that the weight gain with TZDs is associated with an increase in subcutaneous adipose tissue and either no change or a concomitant decrease in visceral fat (reviewed by Larsen et al.) [7]. Since about 90% of type 2 diabetics are obese, treatment with agents that exacerbate obesity is clearly suboptimal. In addition administration of TZDs is often accompanied by an increase in plasma volume [8] and therefore fluid retention is another potential cause of increased body weight.

Edema is a prominent problem in patients taking TZDs particularly those who are also taking insulin or sulfonylureas, and TZD treatment has been linked to an increased incidence of congestive heart failure [8, 9]. Diabetic macular edema has also been recently associated with glitazone use [10]. Because of these serious concerns, several PPAR agonists have failed to progress to FDA approval. A number of glitazars have been terminated in late stage clinical trials because of serious side effects and/or carcinogenesis-related issues including Novo Nordisk's ragaglitazar, GlaxoSmithKline's farglitazar, Merck's MK-767, Takeda's TAK559, and more recently Bristol-Myers Squibb's muraglitazar (Pargluva) and AstraZeneca's tesaglitazar (Galida). Such a high attrition rate emphasizes the critical need for the discovery and characterization of alternative PPAR modulators that would retain the antidiabetic properties while avoiding the side effects.

Starting less than 10 years ago, several TZD-like and non-TZD-like partial PPAR-*γ* agonists that display insulin-sensitizing activity associated with lower stimulation of adipogenesis were described, leading to the emergence of the concept of selective PPAR-*γ* modulators or SPPAR*γ*Ms. This concept is reminiscent of the SERM concept that proposes that different estrogen receptor ligands can have different agonist or antagonist properties depending on the cell context and the specific target gene in question [11, 12]. SPPAR*γ*Ms bind in distinct manners to the ligand-binding pocket of the PPAR-*γ* receptor, leading to differential cofactor displacement and recruitment to the receptor, ultimately resulting in tissue and promoter-selective gene expression. A compound identified by the former Glaxo-Welcome, GW0072, one of the first SPPAR*γ*Ms described in the literature, helped to unravel the partial agonist binding mode. All small molecule PPAR-*γ* full agonists share a common binding mode, in which the acidic head groups bind with 3 amino acid residues (Y473, H449, and H323) within the ligand-binding pocket. These interactions stabilize a charge clamp between the C-terminal activation function 2(AF-2) helix and a conserved lysine residue on the surface of the receptor, through which coactivator proteins are recruited to the receptor [13]. GW0072 was shown to bind to PPAR-*γ* in a unique manner, such that it does not directly interact with the AF-2 helix. Compared to full agonists, the differential binding mode of GW0072 resulted in a differential biological profile that included partial receptor transactivation and reduced ability to recruit specific cofactors and inhibition of adipocyte differentiation [14–16].

The ability to recruit differentially certain cofactors, that is, NR coactivators or corepressors to the PPAR receptor, appears to be the hallmark of the SPPAR*γ*Ms. This likely results in a tissue-specific and promoter-selective expression of a favorable panel of target genes [14, 16–18]. Based on their in vitro and/or in vivo actions, coactivators have been grouped into “adverse” or “beneficial” regarding their proadipogenic or insulin-sensitizing effects. Adverse coactivators include DRIP205/TRAP220 and TIF2. DRIP205/TRAP220-deficient embryonic fibroblasts lack the ability to undergo adipogenesis while TIF2 knockout mice are resistant to diet-induced obesity and are more insulin-sensitive. In contrast, beneficial co-activators include SRC1, as highlighted by the phenotype of SRC1-deficient mice which have reduced energy expenditure and are prone to obesity [12, 19].

Although several PPAR-*γ* agonists have been classified as SPPAR*γ*Ms, the majority of these synthetic ligands remain to be characterized at the molecular level or need to be evaluated in in vivo preclinical models in terms of weight gain. The published characteristics of several SPPAR*γ*Ms have been recently reviewed by others [12, 16, 48, 50] and are summarized in table 1. This review concentrates on the most recent developments in the SPPAR*γ*M arena, including metaglidasen/halofenate, PA-082, and the angiotensin receptor antagonists, recently characterized as a new class of selective PPAR-*γ* modulators.

## 2. HALOFENATE AND METAGLIDASEN: TWO SPPAR*γ*M WITH CLINICAL PROOF OF CONCEPT

Halofenate is a racemic mixture of (−)- and (+)-(2-aceto-aminoethyl [4-chlorophenyl] [3-trifluoromethylphenoxy] acetate). It was tested clinically in the 1970's as a hypolipidemic and hypouricemic agent. In addition to triglyceride and uric acid lowering, significant decreases in fasting plasma glucose were observed in type 2 diabetics. A recently published study demonstrates that halofenate acts as a SPPAR*γ*M [45]. In vivo, halofenate is administered as a prodrug ester, which is rapidly and completely modified to its mature circulating free acid form, halofenic acid (HA). In vitro, HA directly binds to PPAR-*γ* and selectively activates PPAR-*γ* with partial agonism in gene reporter assays (maximal activity at ∼10–15% of the maximal activity of rosiglitazone). HA is also capable of fully antagonizing the activity of the full agonist rosiglitazone. Cofactor recruitment studies reveal that HA effectively displaces the corepressors NCoR and SMRT but is unable to efficiently recruit coactivators (p300, CBP, and DRIP205/TRAP220). HA also displays weak adipogenic activity in human adipocytes and selectively modulates PPAR-*γ* responsive genes in 3T3-L1 adipocytes. Compared with rosiglitazone, HA is unable to efficiently induce genes involved in fatty acid storage and transport, such as FABP4, CD36, GyK, and PEPCK. In vivo, halofenate possesses acute antidiabetic properties in diabetic *ob/ob* mice. Compared with rosiglitazone, long-term treatment of obese Zucker (*fa/fa*) rats with halofenate has comparable insulin sensitization efficacy in the absence of body weight increases. Overall, these in vitro and preclinical data support the concept of halofenate as a novel SPPAR*γ*M.

Metaglidasen (formerly MBX-102) is the (−) enantiomer of halofenate which is currently in Phase II clinical development as an oral glucose-lowering agent for the treatment of type 2 diabetes. In vitro and in vivo preclinical studies revealed that metaglidasen, like halofenate, behaves as a SPPAR*γ*M with antidiabetic and hypolipidemic activity in multiple diabetic and insulin-resistant rodent models [46]. Compared to full PPAR-*γ* agonists, metaglidasen acts as a partial PPAR-*γ* agonist/antagonist that interacts with PPAR-*γ* in a distinct manner. The key amino acid, Tyr473, required for the binding between full agonists to human PPAR-*γ* is not required for metaglidasen activity. Metaglidasen also shows the lack of ability or weak ability to recruit coactivators, including CBP, DRIP205/TRAP220, and p300. Consistently, when compared to rosiglitazone, metaglidasen shows moderate ability to promote adipogenesis and displays largely attenuated induction of PPAR-*γ* target genes involved in fatty acid uptake, synthesis, and storage in primary human adipocytes and mouse 3T3-L1 adipocytes. In vivo, metaglidasen lowers plasma glucose levels in multiple diabetic rodent models (*db/db* mice and ZDF rats) to comparable levels seen with full agonists without causing significant body weight gain, heart weight [46, 47], or plasma volume expansion (unpublished data), a parameter believed to contribute to edema. These observations further support the SPPAR*γ*M concept, confirming the feasibility to separate efficacy and side effects such as edema and weight gain. With respect to edema, thiazolidinediones have been recently reported to expand body fluid volume through PPAR-*γ* stimulation of ENaC-mediated renal salt absorption [51, 52]. Determining if metaglidasen lacks the ability to stimulate increased amiloride-sensitive Na^(+)^ absorption would therefore be important. Consistent with the preclinical findings reported above, in insulin-treated type 2 diabetic patients, metaglidasen appears to have comparable efficacy to the marketed TZDs Actos (pioglitazone) and Avandia (rosiglitazone) while avoiding the limiting side effects of weight gain and edema [53]. These results position metaglidasen as an optimized SPPAR*γ*M with an improved safety profile in comparison to these TZDs.

## 3. A NOVEL PROMISING CLASS OF SPPAR*γ*M: PA-082, A KEY TO UNDERSTANDING THE DISSOCIATION BETWEEN WEIGHT GAIN AND INSULIN SENSITIZATION?

Researchers from Roche have recently described an isoquinoline derivative PA-082 that behaves as a novel partial agonist of the PPAR-*γ* receptor [14]. In cell-based reporter assays, PA-082 was capable of transactivating PPAR-*γ* to about 40% of the level achieved with rosiglitazone. Interestingly this partial agonism was mirrored in its ability to cause partial recruitment of some but not all coactivators to PPAR-*γ*. Using a FRET-based in vitro system, the authors demonstrated that PA-082 elicited a partial recruitment of an LXXLL peptide derived from SRC1, TIF2, and SRC3 to the PPAR-*γ* ligand-binding domain but full recruitment of the LXXLL peptide derived from PGC1-*α*. Importantly this selective recruitment of PGC1-*α* was also observed with the structurally unrelated partial agonists GW0072 and FMOC-L-Leu but not with full agonists that recruited all peptides equally. Preferential recruitment of PGC1-*α* might therefore be a universal hallmark of partial agonists. When compared to the full agonist rosiglitazone, PA-082 prevented triglyceride accumulation during de novo adipogenesis of C3H10T1/2 cells and was also able to antagonize rosiglitazone-induced lipid accumulation. In spite of the partial PPAR-*γ* agonism, PA-082 enhanced insulin-stimulated glucose uptake in adipocytes as well as rosiglitazone suggesting that PA-082 may act to improve whole body glucose disposal without increasing adipose mass. An interesting difference between rosiglitazone and PA-082 was revealed by the observation that in adipocytes PA-082 was more effective than rosiglitazone in preventing insulin resistance induced by TNF*α*. The crystal structure of PA-082 bound to PPAR LBD complexed with LXXLL peptide from SRC1 was also solved. Not surprisingly for a partial agonist, PA-082 did not interact with helix 12, its binding occurring in a part of the binding pocket formed by helices 3, 5, and 7, a site almost identical to that occupied by GW0072 [15]. No preclinical in vivo data are currently available for this compound.

## 4. ANGIOTENSIN RECEPTOR ANTAGONISTS: A NOVEL APPROACH TO ADDRESS THE MULTIFACTORIAL COMPONENTS OF THE METABOLIC SYNDROME?

Recently, angiotensin receptor blockers (ARBs) were reported to have selective PPAR-*γ* modulating activity [41–43, 54, 55]. Among the commercially available ARBs, structurally unique telmisartan appears to be the most potent in terms of PPAR-*γ* activation when tested at concentrations typically achieved in plasma with conventional oral dosing. A growing body of data indicates that telmisartan is a SPPAR*γ*M. In cell-based gene reporter assays, telmisartan behaves as a partial agonist of PPAR-*γ*, giving ∼30% of the maximal PPAR-*γ* activation by full agonist rosiglitazone [41]. Molecular modeling of telmisartan in the PPAR-*γ* ligand-binding domain reveals a different binding mode between telmisartan and rosiglitazone. Specifically, the superimposition of telmisartan on the cocrystal structure of rosiglitazone and PPAR-*γ* showed that telmisartan, like other partial agonists including GW0072 and nTZDpa [15, 21], does not appear to make direct contact with the activation function helix (AF-2). Interaction with the AF-2 helix has been shown to be responsible for receptor stabilization and activation by full agonists of PPAR-*γ* [13]. The lack of interaction of telmisartan with the AF-2 helix likely explains its inability to fully activate the receptor. This differential binding of telmisartan to PPAR-*γ* produced a distinct conformational change compared with rosiglitazone as assessed using a protease protection assay [42]. This in turn results in selective cofactor binding, with the absence of TIF2 recruitment and an attenuated release of the nuclear receptor corepressor NCoR compared with rosiglitazone as assessed by GST pulldown and FRET assays. Differential gene expression profiles by telmisartan versus rosiglitazone were also seen in adipocytes. Compared with rosiglitazone, telmisartan treatment resulted in attenuated induction of genes involved in FA transport and TG storage, including GyK and CD36. Although telmisartan was able to induce adipocyte differentiation [41, 56], the induction was relatively modest compared with full agonists. This is consistent with previous reports showing that other partial agonists of PPAR-*γ* are relatively weak stimulators, or even inhibitors, of adipogenesis [15, 21, 29]. These in vitro data suggest that telmisartan has the potential to lead to less weight gain than the full agonists. This was recently confirmed in vivo. Experiments using diet-induced obese mouse models showed that telmisartan improved insulin sensitivity without causing weight gain [42, 44]. In one study, 10 weeks of telmisartan treatment significantly reduced fasting plasma insulin and glucose levels and improved glucose tolerance and insulin sensitivity. In terms of body weight gain and body fat content, compared with mice treated with vehicle or pioglitazone, mice treated with telmisartan had significantly less weight gain and decreased body fat content in absence of change in food intake [42]. Similar results were reported in a second study of telmisartan treatment for 14 days in diet-induced obese mice. While improving the hyperglycemia, hyperinsulinemia and hypertriglyceridemia, telmisartan treatment attenuated the diet-induced weight gain and decreased the weight of visceral adipose tissue without affecting food intake. Furthermore, telmisartan treatment was also accompanied with increased adiponectin mRNA in visceral white adipose tissue and the serum adiponectin level, reduced the serum level of resistin, increased UCP1 mRNA in brown adipose tissue, and increased oxygen consumption [44]. This suggests that telmisartan treatment may prevent the development of obesity and related metabolic disorders by altering the levels of adiponectin, resistin, and uncoupling protein 1 in these mice. Telmisartan represents a new class of SPPAR*γ*M with in vivo preclinical evidence of maintaining insulin sensitization efficacy while lacking of or preventing weight gain. Although it is unclear yet how much of the efficacy seen is contributed by the attenuated body weight gain and decreased fat mass, preclinical results indicate that telmisartan may be used for treatment of metabolic syndrome and prevention of obesity including visceral obesity.

Whether telmisartan has clinical efficacy in terms of insulin sensitization remains an open question. Several recent studies support the view that telmisartan exerts beneficial effects on lipid and glucose metabolism that involves more than its ability to block the angiotensin II receptor. In an open label post-marketing surveillance study, telmisartan treatment of patients with diabetes (40–80 mg/day in 3642 patients for 6 months) reduced serum glucose and TG compared with baseline [57]. In a randomized, parallel-group study with 40 patients, telmisartan treatment (80 mg/day for 3 months) reduced fasting plasma glucose, insulin resistance (HOMA-IR), and glycated hemoglobin compared with baseline, whereas losartan treatment had no significant effect on any of these parameters [58]. Others angiotensinogen receptor antagonists including losartan, eprosartan, valsartan, and candesartan have also been investigated. In a randomized double-blind, placebo-controlled study with 119 patients, telmisartan treatment (40 mg/day for 12 months), but not eprosartan treatment, reduced plasma total cholesterol, LDL cholesterol, and TG compared with placebo. No change in BMI or glucose metabolism was observed in any group [59]. In a recent study in which valsartan or candesartan were replaced with telmisartan in hypertensive patient with diabetes, the switch to telmisartan was associated with significant reductions in plasma insulin, serum TG, serum CRP levels, as well as increases in serum adiponectin [60]. Telmisartan also reduced serum insulin levels and improved insulin sensitivity as assessed by the homeostasis model in hypertensive nondiabetic patients [61]. Overall, compared with full PPAR-*γ* agonists, the magnitude of the sensitizing effect observed with telmisartan appeared weaker. In terms of adverse effects, no peripheral edema or fluid retention was observed. So far no comprehensive clinical study has evaluated the effects of telmisartan on body weight or adiposity and therefore this remains to be clarified.

## 5. SUMMARY AND FUTURE DIRECTIONS

The in vitro/in vivo data originating from several newly described SPPAR*γ*Ms validate the SPPAR*γ*M concept in term of differential receptor binding, selective cofactor recruitment, and subsequent selective gene expression regulation. The inability to recruit adipogenic cofactors (such as TIF2), the attenuated adipogenic gene expression profile, and the attenuated adipocyte differentiation activity of these SPPAR*γ*Ms are consistent with their lack of weight gain in preclinical models. It is still unclear if the ability to recruit energy expenditure prone cofactor PGC1-*α* is a common characteristic of SPPAR*γ*Ms. Nevertheless, the recruitment of PGC1-*α* may provide a partial explanation in term of their ability to increase UCP1 levels, energy expenditure, and for their antiobesity effects. At this point, the predictive value of interactions between PPAR-*γ* and other coactivators remains uncertain and additional studies using various SPPAR*γ*Ms are needed to further our understanding of these complex interactions. The key questions are does the optimal SPPAR*γ*M already exist? If not, what would the ideal profile of such an optimal SPPAR*γ*M be? And can we rationally design preclinical strategies to identify it? There is no doubt that comparison of the differential cofactor recruitment and selective gene expression regulation by various SPPAR*γ*Ms will generate a wealth of information that will further our mechanistic understanding of SPPAR*γ*M biology. Recent clinical data obtained with metaglidasen confirm that SPPAR*γ*Ms can maintain efficacy while lacking the typical side effects such as edema and weight gain, supporting the concept that the SPPAR*γ*M represents the next generation of insulin sensitizers.

## Figures and Tables

**Table 1:** Investigational SPPAR*γ*M ligands for the treatment of type 2 diabetes.

Compound	Transcriptional activity (% full agonist)	Adipogenesis (versus full agonists)	Body weight gain (versus full agonist)	Cofactors recruitment capacity (versus full agonists)	Development stage	Refs.

GW0072	∼20–40%	Partial	No data	Decreased (CBP, SRC1, TIF2, SCR3)	Preclinical	[14, 15]
				Similar (PGC1-*α*)		
				Lack of recruitment (NCoR, SMRT)		

FMOC-L-Leucine	∼40–100%	Partial	None in a week	Decreased (p300, PGC1-*α*)	Preclinical	[14, 20]
				Lack of recruitment (CBP, TIF2, SCR3)		
				Inconsistent data for SRC1		

nTZDpa	∼25%	Partial	Decreased	No data	Preclinical	[21]

L-764406	∼25%	Partial	No data	Decreased (CBP)	Preclinical	[22]

YM440	∼10–80% (CV-1)	Minimal	None	Similar (p300, SRC1)	Phase II discontinued	[23–25]
	100% (hepG2)					

DRF-2593 (balaglitazone)	∼78%	Partial	Moderate	No data	Phase II	[26–28]

MCC555 (netoglitazone)	∼50–100%	Similar	None	Decreased (CBP, SRC1)	Phase II	[29–31]
				Similar (SMRT)		

CLX-0921	100%	Partial	None in 9 days	Recruit CBP (no data in comparison with full agonists)	Preclinical discontinued	[32]

Compound 24 (benzoyl-2-methyl indole)	21%	Partial	Minimal	No data	Preclinical	[33]

Compound 12 (N-benzyl-indole)	24%	Minimal	No data	No data	Preclinical	[34]

Compound 5 (aryl indole-2-carboxylic acid)	31%	No data	Minimal in 11 days	No data	Preclinical	[35]

FK-614	∼65%	Similar	Similar	Decreased (CBP, SRC1)	Preclinical	[36–39]
				Similar (PBP, PRIP, PGC1-*α*, NCoR, SMRT)		

KR-62980	∼30%	None to partial	Decreased	Decreased (AIB-1, SRC1, TRAP220)	Preclinincal	[40]
				Similar (TIF2, p300)		

Telmisartan (ARBs)	∼30%	Partial	Decreased	Decreased (NcoR release)	Marketed	[41–44]
				Similar (DRIP205)		
				Lack of recruitment (TIF2)		

PA-082	∼40%	Partial	No data	Decreased (SRC1, TIF2, SCR3)	Preclinical	[14]
				Similar (PGC1-*α*)		

Halofenate/metaglidasen	∼10–15%	Partial	Decreased	Decreased (CBP, P300, TRAP220)	Phase II (metaglidasen)	[45–47]
				Similar (NCoR, SMRT)		

AMG-131	Cell type	Minimal	No data	Decreased (DRIP205)	Phase II	[48, 49]
T-131	dependent			Increased association (NCoR)	discontinued	
